# Unraveling the Genomic-Epigenomic Interaction Landscape in Triple Negative and Non-Triple Negative Breast Cancer

**DOI:** 10.3390/cancers12061559

**Published:** 2020-06-12

**Authors:** Jiande Wu, Tarun Karthik Kumar Mamidi, Lu Zhang, Chindo Hicks

**Affiliations:** 1 Health Sciences Center, Department of Genetic, Louisiana State University School of Medicine, 533 Bolivar Street, New Orleans, LA 70112, USA; jwu2@lsuhsc.edu; 2 Center for Computational Genomics and Data Science, Departments of Pediatrics and Pathology, University of Alabama–Birmingham School of Medicine, Birmingham, AL 35233, USA; tmamidi@uab.edu; 3 Department of Public Health Sciences, Clemson University, 513 Edwards Hall, Clemson, SC 29634, USA; lz3@clemson.edu

**Keywords:** gene expression, somatic, genomics, epigenomics, interactions, breast cancer

## Abstract

**Background**: The recent surge of next generation sequencing of breast cancer genomes has enabled development of comprehensive catalogues of somatic mutations and expanded the molecular classification of subtypes of breast cancer. However, somatic mutations and gene expression data have not been leveraged and integrated with epigenomic data to unravel the genomic-epigenomic interaction landscape of triple negative breast cancer (TNBC) and non-triple negative breast cancer (non-TNBC). **Methods**: We performed integrative data analysis combining somatic mutation, epigenomic and gene expression data from The Cancer Genome Atlas (TCGA) to unravel the possible oncogenic interactions between genomic and epigenomic variation in TNBC and non-TNBC. We hypothesized that within breast cancers, there are differences in somatic mutation, DNA methylation and gene expression signatures between TNBC and non-TNBC. We further hypothesized that genomic and epigenomic alterations affect gene regulatory networks and signaling pathways driving the two types of breast cancer. **Results**: The investigation revealed somatic mutated, epigenomic and gene expression signatures unique to TNBC and non-TNBC and signatures distinguishing the two types of breast cancer. In addition, the investigation revealed molecular networks and signaling pathways enriched for somatic mutations and epigenomic changes unique to each type of breast cancer. The most significant pathways for TNBC were: retinal biosynthesis, BAG2, LXR/RXR, EIF2 and P2Y purigenic receptor signaling pathways. The most significant pathways for non-TNBC were: UVB-induced MAPK, PCP, Apelin endothelial, Endoplasmatic reticulum stress and mechanisms of viral exit from host signaling Pathways. **Conclusion**: The investigation revealed integrated genomic, epigenomic and gene expression signatures and signing pathways unique to TNBC and non-TNBC, and a gene signature distinguishing the two types of breast cancer. The study demonstrates that integrative analysis of multi-omics data is a powerful approach for unravelling the genomic-epigenomic interaction landscape in TNBC and non-TNBC.

## 1. Introduction

Despite remarkable progress in screening and patient management, breast cancer remains the most commonly diagnosed non-skin cancer and the second leading cause of cancer related death among women in the US [1]. In 2019, 268,600 women were newly diagnosed with breast cancer, and 41,760 women died from the disease in the US [1]. Breast cancer is a heterogeneous disease encompassing many types and subtypes. A majority of breast cancers are non-triple negative breast cancers (non-TNBC). These types of breast cancer are characterized by less aggressive clinical behavior, good prognosis, low recurrence and higher survival rates [2,3]. Importantly, these cancers respond to endocrine and targeted therapies [2,3].

However, a significant proportion of breast cancers ~15–20% are triple negative breast cancers (TNBC), the most aggressive and lethal form of breast cancer [2,3]. TNBC are defined as tumors that lack expression of the estrogen receptor (ER), progesterone receptor (PR), and human epidermal growth factor receptor (HER-2) amplification [2,3]. TNBC is characterized by poorer prognosis, higher recurrence rates and worse overall survival rates than non-TNBC [2,3]. Most notably, unlike non-TNBC, currently there are no effective targeted therapies for TNBC, cytotoxic chemotherapy remains the only effective therapeutic modality [2,3]. Accumulating evidence from both epidemiologic and clinical studies has revealed that TNBC has worse outcomes and survival rates than non-TNBC [4,5,6,7,8,9,10,11]. However, the molecular mechanisms underlying the etiological and biological differences between the two types of breast cancer have not been well characterized. Thus, a key knowledge gap and critical unmet medical need pivot around: (i) understanding the molecular mechanisms underlying the biological differences between TNBC and non-TNBC; (ii) the discovery of clinically actionable molecular markers and potential targets for the development of novel therapeutics unique to each type of breast; and (iii) the identification of biomarkers distinguishing the two types of breast cancer that could be used to identify and stratify patients to guide treatment decisions.

Our understanding of the molecular mechanisms underlying the biological differences between TNBC and non-TNBC and how best to treat them is hampered by the biological and etiological complexity of the two diseases. Both types of breast cancer are heterogeneous diseases, each involving many subtypes, and their development and progression involves a complex interplay between genomic and epigenomic alterations and many environmental perturbations. These complex arrays of interacting factors likely affect entire molecular networks and signaling pathways, which in turn drive the two types of breast cancer. Integrative data analysis combining genomic, epigenomic and gene expression data has the promise to increase our understanding of the biological mechanisms driving the two types of breast cancer, and to identify clinically actionable molecular markers and targets to guide therapeutic decisions.

Advances in microarray technology have enabled the molecular classification of types and subtypes of breast cancer using transcription profiling [12,13,14]. Evidence from these primary studies have revealed differences in the molecular portraits of TNBC and non-TNBC [12,13,14]. Transcription profiling has enabled development of two prognostic signatures, the PAM50 [15,16] and the MammaPrint [17,18], which have proven useful for prognostic purposes in the clinic [15,16,17,18]. However, although these primary analyses have made great strides in deciphering the molecular basis of breast cancer and enabled development of prognostic signatures, they have been unsuccessful in determining how genomic and epigenomic factors interact and cooperate to drive each type of breast cancer.

Over the last decade, rapid development in next generation sequencing technologies has brought considerable advances in large-scale sequencing of cancer genomes, including breast cancer [19,20,21]. These advances have enabled development of comprehensive catalogues of somatic mutations and epigenomic signatures of breast cancer. In addition, these advances have led to an expanded molecular classification and increased our understanding of the molecular taxonomy of breast cancer. Traditionally, the analyses of somatic, epigenomic and gene expression data in different types of breast cancer have been conducted as separate research endeavors. Recently, we reported a novel approach integrating somatic mutation with DNA methylation information using gene expression data in TNBC [22]. This investigation showed that somatic mutated genes harbor epigenomic alterations, and that epigenetic changes affect gene expression [22]. However, to date, information on somatic mutations has not been leveraged and integrated with DNA methylation data to unravel the possible oncogenic interactions between genomic and epigenomic alterations in TNBC and non-TNBC. The objectives of this study were to: (i) define integrated genomic, epigenomic and gene expression profiling signatures unique to TNBC from non-TNBC; (ii) discover a signature distinguishing the two types of breast cancer; and (iii) map the possible oncogenic interactions between genomic and epigenomic alterations in TNBC and non-TNBC, using network and pathways analysis. Our working hypothesis was that within breast cancers, there are differences in somatic mutation, DNA methylation and gene expression signatures between TNBC and non-TNBC. We further hypothesized that genomic and epigenomic alterations affect gene regulatory networks and signaling pathways driving the two types of breast cancer. We addressed these hypotheses using an integrative genomics data analysis approach, combining information on somatic, epigenomic and gene expression data on TNBC and non-TNBC from The Cancer Genome Atlas (TCGA).

## 2. Materials and Methods

### 2.1. Overall Project Design and Execution Strategy

**Figure 1:**
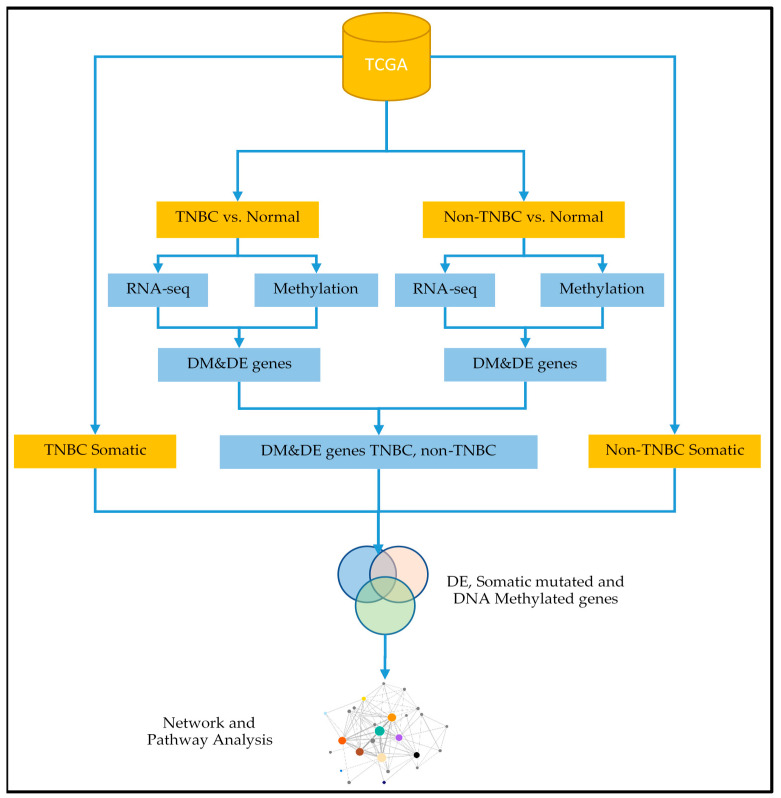
Project design and integrated analysis workflow integrating somatic mutation, DNA methylation and gene expression data leveraged with network, pathway and functional analysis to unravel the genomic-epigenomic interaction landscape in triple negative breast cancer (TNBC) and non-triple negative breast cancer (non-TNBC). DM indicates differentially methylated, DE indicates differentially expressed.

The overall project design and workflow for integrative analyses strategies, integrating somatic, DNA methylation and gene expression data, is presented in Figure 1.

### 2.2. Sources of Genomic and Epigenomic Data

The TCGA data management team has assembled genomic, epigenomic and mutation data, along with clinical information, and has made it available to the cancer research community [19,20] via the Genomics Data Commons [23]. For this study, we used gene expression, DNA methylation (array-based) and somatic mutation data from the TCGA [19]. The data sets were downloaded from the Genomics Data Commons data portal using the data transfer tool [23]. The original gene expression data set was generated using RNA-Seq and consisted of 828 samples, distributed as follows: patients diagnosed with TNBC tumors *n* = 110 samples, patients diagnosed with non-TNBC tumors *n* = 605 samples and controls *n* = 113 samples (Table 1). The original gene expression data matrix included 60,484 probes (Table 1).

The original DNA methylation (array-based) data set consisted of 763 samples, distributed as follows: patients diagnosed with TNBC tumors *n* = 83 samples, patients diagnosed with non-TNBC tumors *n* = 597 samples and controls *n* = 83 samples (Table 1). DNA methylation data was generated using Illumina HumanMethylation450 BeadChip (Illumina Inc. San Diego, CA, USA). This chip has been widely used for quantifying DNA methylation and has been validated [24]. The DNA methylation data matrix included 485,578 probes (Table 1). We processed and performed data quality control (QC) using the HumanMethylation450 evaluation and processing methods [25,26], implemented in the DNA methylation data analysis pipeline developed in our lab [22]. The data was further processed to remove batch effects prior to analysis [22,26]. Considering the convolution of biological and technological variability and the signal bias between the Infinium I and II probe design types, the data was further quality controlled to correct for type of probe design [27], and to eliminate bias attributable to systematic errors consistent with the Illumina data analysis protocol [25,26]. We used the quantile normalization implemented in R software package [22,24,25,26,27] to normalize the data and calibrated for batch effects consistent with the Illumina data analysis protocol [25,26,27].

The DNA methylation data was derived from the same patient population as gene expression and somatic mutation data. Therefore, following processing and quality control of disparate data sets, we optimized the data matrix for integrated analysis by identifying samples with all three pieces of information. To address this issue, we used clinical information to link the methylation samples with the gene expression samples. After this processing step, we used 83 patients diagnosed with TNBC tumors, 597 patients diagnosed with non-TNBC tumors and 83 controls (Table 1) in the analysis. We then used a barcode structure provided by TCGA to integrate patient-based clinical data with sample-based somatic mutation data, and processed somatic mutation data to identify the number of genes that contain somatic mutations and the number of somatic mutation events for each gene across samples within the tumor and across tumors. In this processing step, we created a catalog of 7659 somatic mutant genes in TNBC and 16,752 somatic mutant genes in non-TNBC for analysis (Table 1). Supplementary Table S1 presents a comprehensive list of TNBC and non-TNBC somatic mutation genes and the number of somatic mutation events for each gene.

### 2.3. Bioinformatics Analysis of Disparate Omics Data

Figure 1 shows the data processing and analysis workflow. In this analysis, we focused on gene expression and DNA methylation data, analyzing them separately for each type of breast cancer. Note that only samples with both gene expression and DNA methylation were used in these analyses. We performed QC and noise reduction on the original gene expression data matrix to remove rows with insufficient information or missing data. The filtering was imposed such that each row had at least ≥30% data. After this QC step, the data matrix for TNBC reduced to 166 samples (83 TNBC tumors samples and 83 controls) with 28,084 probes used in the analysis. Likewise, using the same data processing and QC for non-TNBC reduced the data matrix to 680 samples distributed as (597 non-TNBCs tumor samples and 83 controls) with 27,218 probes used in the analysis. The quality-controlled data sets were normalized using the LIMMA Bioconductor package implemented in R [28]. The probe IDs were matched with gene symbols using the Ensemble database [29]. Gene expression data from RNA-Seq was quantified in Transcripts Per Kilobase Million (TPM), and was first log2(TPM + 1) transformed using the standard protocol for RNA sequence data normalization methods [30].

Using quality controlled normalized data sets, we compared gene expression levels between TNBC tumors and control samples, to discover a signature of significantly differentially expressed genes in TNBC. Likewise, we compared gene expression levels between non-TNBC tumors and control samples, to discover a signature of significantly differentially expressed genes in non-TNBC. Additionally, we performed supervised analysis comparing gene expression levels between TNBC and non-TNBC, to identify a signature of significantly differentially expressed genes distinguishing the two types of breast cancer. These robust and unbiased analyses enabled the discovery of all differentially expressed genes, including somatic mutated ones. Gene expression analysis was performed using the Bioconductor R-package LIMMA [28]. Throughout these analyses, we used the false discovery rate (FDR) method [31] to adjust the *p*-value for multiple hypothesis testing. In addition, for each analysis, we calculated the log2 Fold Change (Log2 FC), defined as the median of gene expressed minus the gene expression value for each gene.

For each type of breast cancer, genes were ranked on adjusted *p*-value, FDR and LogFC, and significantly (*p* < 0.05) differentially expressed genes were identified and selected. Note that significant differential expression provides proof of association of these genes with the types of breast cancer. The discovered significantly differentially expressed genes were evaluated for the presence of somatic mutations to discover signatures of somatic mutated genes, which are significantly differentially expressed in each type of breast cancer and a signature of somatic mutated genes distinguishing the two types of breast cancer. Somatic mutated genes were further quantitatively assessed for the number of mutation events. A gene was considered highly mutated if it contained at least ≥3 mutation events. A gene was considered differentially mutated if it was mutated only in one type of breast cancer, or if the mutations found in one type of breast cancer were not found in the other type.

Likewise for DNA methylation data, we performed QC to remove batch effects [22] and noise reduction on the original data set to remove rows with insufficient information or missing data, using the same filtering threshold of each row having at least ≥30% data. After this QC step, the data matrix for TNBC reduced to 166 samples (83 TNBC tumors samples and 83 controls), with 383,119 probes used in the analysis. Using the same data processing and QC for non-TNBC reduced the data matrix to 680 samples distributed as (597 non-TNBCs tumor samples and 83 controls), with 365,947 probes used in the analysis. The quality-controlled data sets were normalized using quantile normalization applied to the LIMMA Bioconductor package implemented in R [22,27,28].

Using the quality controlled normalized DNA methylation data set, we compared DNA methylation profiles using beta-values between TNBC tumor and control samples and between non-TNBC tumor and control samples, to discover signatures of significantly (*p* < 0.05) differentially methylated genes associated with each type of breast cancer [22]. These analyses were accomplished using the methods for analysis of DNA methylation data in the Illumina protocol [24,25,26], implemented in our pipeline [22]. In addition, we performed supervised analysis comparing DNA methylation profiles between TNBC and non-TNBC, to identify a signature of significantly differentially methylated genes between the two types of breast cancer. We used the LIMMA package to identify differential methylated CpG sites [22,28]. For all the analyses we used the FDR [31] to control for multiple hypothesis testing. Genes were ranked on adjusted *p*-values, FDR and LogFC. We used the Ensemble Biomart database to link CpG sites with gene symbols [22,32]. Significantly differentially methylated genes were further quantitatively evaluated for the number of differentially CpG sites. A gene was considered highly differentially methylated if it contained ≥3 methylation events. A gene was considered differentially methylated if it was aberrantly methylated only in one type of breast cancer, or if the CpG sites found in one type of breast cancer were not found in the other type.

For each type of breast cancer, genes were ranked on adjusted differential methylation *p*-values, number of CpG sites per gene, FDR and LogFC. The discovered significantly differentially methylated genes were evaluated for the presence of somatic mutations, to discover the signatures of somatic mutated genes that are significantly differentially methylated in each type of breast cancer, and a signature of differentially methylated somatic mutated genes, distinguishing the two types of breast cancer. Somatic mutated genes were further quantitatively assessed to determine the number of mutation events. The same criteria as in gene expression was used for identifying highly mutated and differentially mutated genes.

### 2.4. Integrative Analysis of Diverse Omics Data

We used a systems level integration to investigate potential oncogenic interactions between genomic and epigenomic alterations in TNBC and non-TNBC. The interplay between genomic and epigenomic alterations may happen both in *cis* and in *trans*, so we performed multiple levels of integration. At level 1 integration, we integrated information on differentially expressed genes with information on differentially methylated genes in TNBC and non-TNBC. Here, we sought to: (i) discover the signatures of differentially expressed genes, which are also differentially methylated (integrated signature) and uniquely associated with each type of breast cancer; and (ii) to discover a signature of significantly differentially expressed genes which were also significantly differentially methylated distinguishing the two types of breast cancer. At level 2 integration, we combined information on differentially methylated, expressed and somatic mutated in TNBC and non-TNBC. Here; we sought to: (i) discover the integrated signatures of differentially expressed genes, which are also differentially methylated and differentially mutated, uniquely associated with each type of breast cancer; and (ii) to discover an integrated signature of significantly differentially expressed genes, which are also differentially methylated and differentially mutated, distinguishing the two types of breast cancer. For each integrated signature, we conducted a quantitative assessment of somatic mutations per gene, within and between the two types of breast cancer, to assess the uniqueness and the differences in mutation burden between TNBC and non-TNBC.

The final levels of integrative analysis involved network (level 3), pathway (level 4) and functional (level 5) analysis of genes containing somatic and epigenomic alterations. Here, we sought to unravel the possible oncogenic interactions between genomic and epigenomic in TNBC and non-TNBC. We performed network and pathway prediction using the Ingenuity Pathway Analysis (IPA) Software (Qiagen Inc., Hilden, German) [33] to identify the molecular networks and signaling pathways enriched for somatic and epigenomic alterations in each type of breast cancer. Somatic mutated genes, which were differentially methylated and transcriptionally associated with each type of breast cancer were mapped onto networks and canonical pathways using IPA. For network analysis, a Z-score was computed, whereas for pathway prediction, the –log(*p*-values) were computed and used as the metrics for correctly assigning the genes to the correct networks and pathways, respectively. The FDR was used to correct for type I and type II errors, to ensure the reliability of correctly predicting the pathways. We performed GO [34] analysis using the IPA database to functionally characterize the discovered genes into the molecular functions, biological process and cellular components in which they are involved.

## 3. Results

### 3.1. Gene Expression and DNA Methylation Sigantures Associated with TNBC and Non-TNBC

We performed whole transcriptome and whole methylome analysis to identify gene expression and DNA methylation profiling signatures uniquely associated with TNBC and non-TNBC and signatures distinguishing the two types of breast cancer. We hypothesized that the molecular perturbation and aberrant DNA methylation in tumors from patients diagnosed with TNBC or non-TNBC and control samples could lead to measurable changes distinguishing patients diagnosed with TNBC tumors from control samples, and patients diagnosed with non-TNBC tumors from control samples. To address these hypotheses, we performed whole transcriptome and whole methylome analysis comparing gene expression levels and DNA methylation profiles between tumor and control samples for TNBC and non-TNBC separately.

Whole transcriptome analysis comparing gene expression levels between tumor and control samples in TNBC produced a signature of 15,404 significantly (*p* < 0.05) differentially expressed genes. Comparing gene expression levels between tumor samples and controls in non-TNBC tumors produced a signature of 16,124 significantly (*p* < 0.05) differentially expressed genes. Complete lists of the 15,404 genes associated with TNBC and the 16,124 genes associated with non-TNBC along with estimates of *p*-values, FC indicative of the level of association, as well as their direction of change (up or down regulated) are presented in supplementary Table SE.

Whole methylome analysis comparing DNA methylation profiles between tumor and control samples in TNBC produced a signature of 21,196 significantly (*p* < 0.05) differentially methylated genes. Comparing DNA methylation profiles between patients diagnosed with non-TNBC and controls revealed a signature of 21,415 significantly (*p* < 0.05) differentially methylated genes. Complete lists of the 21,196 genes significantly differentially methylated in TNBC and the 21,415 genes significantly differentially methylated in non-TNBC, along with estimates of *p*-values, FC indicative of the level of association, as well as their direction of change (hyper or hypo methylated), are presented in supplementary Table SM.

### 3.2. Discovery of Integrated DNA Methylation and Gene Expression Signatures

To discover the signatures of significantly (*p* < 0.05) differentially expressed genes, which were also significantly (*p* < 0.05) differentially methylated (herein called integrated signatures) unique to TNBC and non-TNBC, or genomically and epigenomically altered in both types of breast cancer, we performed analysis combining information on significantly (*p* < 0.05) differentially expressed genes and significantly (*p* < 0.05) differentially methylated genes in each type of breast cancer, compared to controls. For TNBC, we combined the 15,404 significantly (*p* < 0.05) differentially expressed genes with the 21,196 significantly (*p* < 0.05) differentially methylated genes. Similarly, for non-TNBC, we combined the 16,124 significantly (*p* < 0.05) differentially expressed genes with the 21,415 significantly (*p* < 0.05) differentially methylated genes. Genes were then sorted by gene symbols to identify the integrated DNA methylation and gene expression signatures unique to each type of breast cancer, and signatures that overlapped the two types of breast cancer.

**Figure 2:**
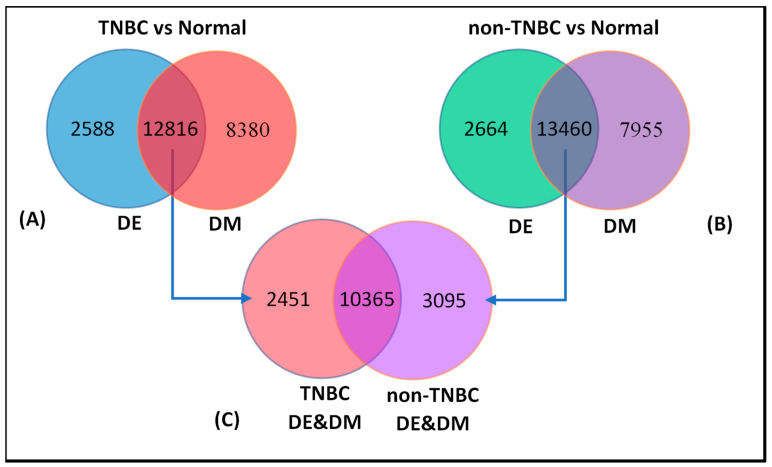
Number of genes significantly (*p* < 0.05) differentially expressed or significantly (*p* < 0.05) differentially methylated in each type of breast cancer (outside intersections), and genes significantly (*p* < 0.05) differentially expressed and significantly (*p* < 0.05) differentially methylated in each type of breast cancer (in the intersection) are shown in (**A**) for TNBC and (**B**) for non-TNBC, respectively. Figure (**C**) shows numbers of significantly (*p* < 0.05) differentially expressed genes that are significantly (*p* < 0.05) differentially methylated unique to each type of breast cancer (outside the interaction), and genes overlapping the two types of breast cancer (see intersections). Please note, as indicated by the arrows, that the numbers in (**C**) are derived from the number intersections of (**A**) and (**B**).

The results of these analyses are summarized in Figure 2. For TNBC, we discovered an integrated DNA methylation and gene expression signature of 12,816 genes. In addition, 2588 genes were significantly (*p* < 0.05) differentially expressed, but not differentially methylated, whereas 8380 genes were significantly (*p* < 0.05) differentially methylated, but not differentially expressed (Figure 2A). For non-TNBC, we discovered an integrated DNA methylation and gene expression signature of 13,460 genes. In addition, we discovered a signature of 2664 significantly (*p* < 0.05) differentially expressed genes, which were not aberrantly methylated and a signature of 7955 significantly (*p* < 0.05) differentially methylated genes, which were not differentially expressed (Figure 2B). These findings confirmed our hypothesis that, within each type of breast cancer, there were genes that were significantly altered in both the transcriptome and the methylome. Supplementary Table S2A,B shows complete lists of all the 12,816 genes in TNBC and 13,460 genes in non-TNBC, respectively, which were significantly (*p* < 0.05) altered in both the transcriptome and the methylome.

Having discovered the signatures of genes altered in both the transcriptome and the methylome in each type of breast cancer (Figure 2A,B intersections), we performed an additional investigation to discover the signatures of genes unique to each type of breast cancer, and a signature of genes genomically and epigenomically altered in both types of breast cancer. We addressed this question by combining the 12,816 genes from TNBC (Figure 2A) with the 13,460 genes from non-TNBC (Figure 2B) with both alterations, and sorted them by gene symbol, expression and methylation *p*-values. The results of this investigation are presented in Figure 2C. The analysis revealed a signature of 10,365 genes genomically and epigenomically altered in TNBC and non-TNBC. In addition, the analysis revealed a signature of 2451 genes genomically and epigenomically altered in TNBC only, and a signature of 3095 genes altered in non-TNBC only (Figure 2C). A complete list of all the 10,365 significantly (*p* < 0.05) differentially expressed genes, which were also significantly (*p* < 0.05) differentially methylated in TNBC and non-TNBC, is presented in supplementary Table S2C. These analyses suggest that for a subset of genes, molecular perturbation and epigenomic changes occur in both types of breast cancer, but at different rates.

### 3.3. Discovery of Epigenomic and Gene Expression Signatures Distinguishing TNBC from Non-TNBC

A critical need in understanding the biology of breast cancer is characterizing the molecular differences between TNBC and non-TNBC tumors. We addressed this issue by testing the hypothesis that genomic and epigenomic (or a combination thereof) alterations differ between TNBC and non-TNBC. Here, we reasoned that, because of the etiological and biological differences between TNBC and non-TNBC, the genomic and epigenomic mechanisms driving the two types of breast cancer may be different. To test this hypothesis, we created and used a new data set containing the 10,365 genes genomically and epigenomically altered in both TNBC and non-TNBC derived from (Figure 2C). We compered their expression levels and DNA methylation profiles between TNBC and non-TNBC.

**Figure 3:**
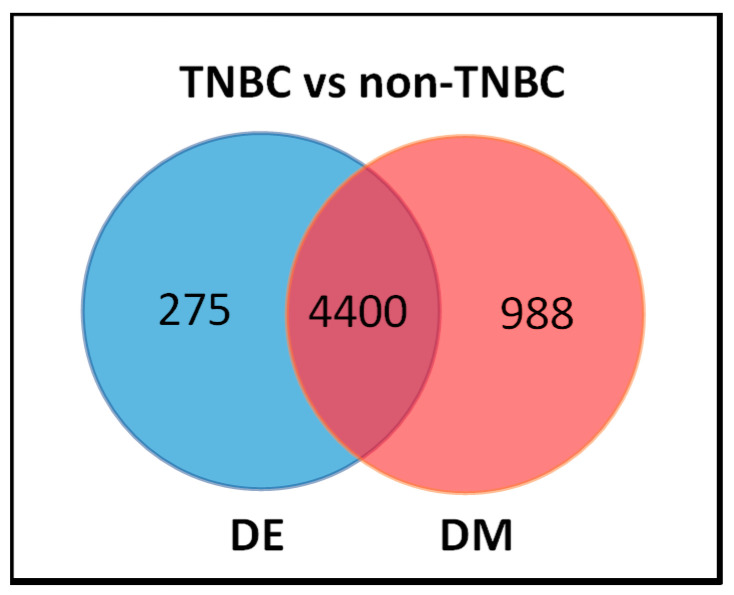
Venn diagram showing the distribution of genes containing both differentially expressed (DE) and differentially methylated (DM), showing the 4400 genes distinguishing TNBC from non-TNBC (in the interaction), 275 differentially expressed (DE) only, and the 988 differentially methylated (DM) only between TNBC and non-TNBC.

The results of this investigation are shown in a Venn diagrams in Figure 3. We discovered a signature of 4675 significantly (*p* < 0.05) differentially altered genes, and a signature of 5388 significantly (*p* < 0.05) differentially methylated genes distinguishing the two types of breast cancer. Interestingly, we also discovered a signature of 4400 genes significantly (*p* < 0.05) differentially in the transcriptome, which were also significantly (*p* < 0.05) differentially methylated, distinguishing TNBC from non-TNBC (Figure 3, intersection). In addition, the analysis revealed a signature of 275 significantly (*p* < 0.05) differentially expressed genes without DNA methylation, and a signature of 988 significantly (*p* < 0.05) differentially methylated genes not differentially expressed (Figure 3) between the two types of breast cancer. This confirmed our hypothesis that an integrative analysis of genomic and epigenomic data could lead to the discovery of a signature of genes altered both in the transcriptome and the methylome, and distinguishing the two types of breast cancer.

To further explore the patterns of gene expression and DNA methylation, we evaluated the 4400 genes (Figure 3). The results showing the distribution of DNA methylation for the top list of genes that were highly significantly differentially altered in the transcriptome and the methylome between TNBC and non-TNBC are presented in Table 2. Only the genes with a high frequency of DNA methylation (DM) sites per gene are presented in the Table. A full list of all the 4400 genes significantly differentially altered in both the transcriptome and the methylome distinguishing the two types of breast cancer is presented in supplementary Table S3A. A full list of all the 5663 genes with a binary pattern of presence and absence is presented in supplementary Table S3B.

Overall, there was significant variation in patterns of DNA methylation, as measured by the distribution of significantly differentially methylated CpG sites and gene expression levels within and between the two subtypes of breast cancer. The observed variation in patterns of gene expression and DNA methylation profiles can be explained partially by the heterogeneity of these diseases. Both TNBC and non-TNBC are inherently heterogeneous types of breast cancer, each comprised of many subtypes of breast cancer. Under such conditions, the observed outcome should be expected. Due to the lack of information on the subtypes of breast cancer in the data sets used in this investigation, we did not address the issue of subtypes, as it was beyond the scope of this study.

### 3.4. Integration of Somatic and Epigenomic Variation

Having discovered an integrated signature of genes altered both in the transcriptome and the methylome distinguishing TNBC from non-TNBC, we conducted further investigations to integrate somatic mutation information. We hypothesized that genes differentially altered in the transcriptome and the methylome harbor somatic mutations. We further hypothesized that there are differences in somatic mutation burden and epigenetic alterations between the two types of breast cancer. Here, we sought to discover the somatic, epigenomic and gene expression signatures unique to TNBC and non-TNBC, and a signature distinguishing the two types of breast cancer. We addressed this issue as follows: first, we evaluated differentially expressed and differentially methylated genes for the presence of somatic mutations, using the 7659 somatic mutated genes in TNBC and the 16,752 genes somatic mutated genes in non-TNBC. We also evaluated the 4400 genes altered in the transcriptome and the methylome distinguishing TNBC from non-TNBC for the presence of mutations, and for differences in mutation burden between the two types of breast cancer. Finally, we performed a quantitative assessment of the differences in somatic mutations and epigenomic alterations between TNBC and non-TNBC.

**Figure 4:**
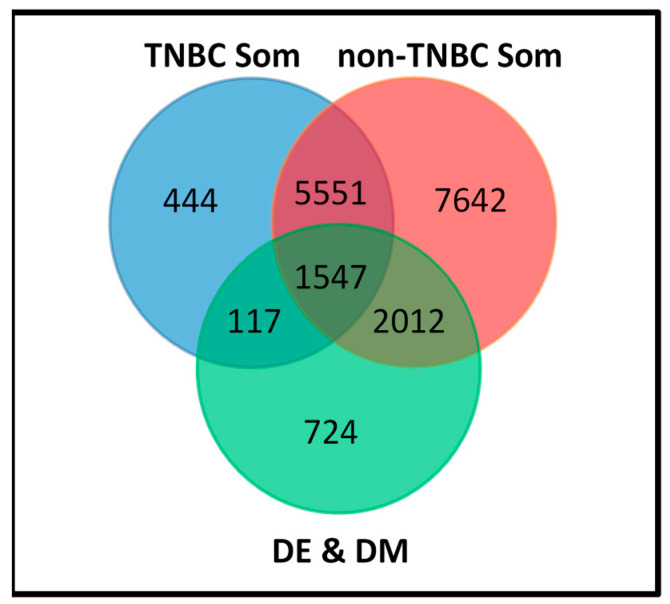
Somatic and epigenomic altered gene signatures unique to TNBC and non-TNBC, signatures common to both types of breast cancer and signatures distinguishing them.

The results of these analyses are summarized in a Venn diagram in Figure 4. Out of the 7659 genes somatic mutated in TNBC, 444 genes contained somatic mutations in TNBC only, and were neither differentially methylated nor differentially expressed between the two types of breast cancer (Figure 4). Out of the 16,752 genes somatic mutated in non-TNBC, 7642 contained somatic mutations in non-TNBC only, and were neither differentially methylated nor differentially expressed between the two types of breast cancer (Figure 4). However, a complete evaluation of all somatic mutated genes for genomic and epigenomic alterations revealed a total of 5551 genes containing somatic mutations in both types of breast cancer, and were neither differentially methylated nor differentially expressed between the two types of breast cancer (Figure 4).

Quantitative evaluation of the somatic mutated genes against the 4400 genes altered in both the transcriptome and the methylome distinguishing TNBC from non-TNBC revealed three gene signatures. Among them were the 117 gene signature containing both somatic and epigenomic alterations unique to TNBC, the 2012 gene signature containing both somatic and epigenomic alterations unique to non-TNBC and the 1547 gene signature containing both somatic and epigenomic alterations, which overlapped the two types of breast cancer (Figure 4). In addition, we discovered a 724 gene signature containing epigenomic alterations without somatic mutations (Figure 4). There was significant variation in both somatic mutation and DNA methylation profiles within and between the two types of breast cancer. As noted earlier in this report, this was expected, because breast cancer is inherently a heterogeneous disease, thus, the observed outcome was expected.

To further assess the patterns of gene expression, DNA methylation and somatic mutation profiles, we evaluated the genes containing all the three variations. Table 3 shows subsets of the top somatic mutated and epigenomically altered genes from the 117 genes unique to TNBC and the 2012 genes unique to non-TNBC, which distinguished the two types of breast cancer. The distributions of methylation sites, somatic mutation evens and adjusted estimates of differential expression and differential methylation *p*-values are also presented in Table 3. Among the 117 genes unique to TNBC, the number of somatic mutation events per gene ranged from one to three, whereas the number of DNA methylation sites or CpG sites ranged from one to 63. Among the 2012 genes unique to non-TNBC, the number of somatic mutation events per gene ranged from one to 33, whereas the number of DNA methylation sites or CpG sites ranged from one to 165. There were significant differences and variation in the number of somatic mutation and DNA methylation events in the same gene in each type of breast cancer. A list of the top most highly somatic mutated and aberrantly methylated genes is presented in Table 3. A full list of somatic mutated genes, which were significantly differentially altered in the transcriptome (DE) and differentially altered in the methylome between TNBC and non-TNBC are presented in Supplementary Table S4. There were significantly differences in somatic, DNA methylation and gene expression variation between TNBC and non-TNBC. The significant differences in DNA methylation and somatic mutation profiles suggest that TNBC and non-TNBC may be amenable for somatic mutation-based and methylation-based classification, or a combination thereof.

Further evaluation of the frequency of somatic mutations in each type of breast cancer revealed higher mutation occurrence in non-TNBC, compared to TNBC (Table 3). In addition, there was an uneven distribution of somatic mutation and epigenomic events. This suggests that somatic and epigenomic alterations are likely to occur at different times during the course of each disease. The results confirm our hypothesis that, for a selected set of genes, there were significant differences in somatic mutation burden, DNA methylation and gene expression levels between TNBC and non-TNBC (Table 3). As noted earlier in this report, the significant variation in somatic mutations, DNA methylation and gene expression levels can partially be explained by the heterogeneous nature of the two types of breast cancer.

### 3.5. Discovery of Gene Regulatory Networks and Biological Pathways

The development and progression of TNBC and non-TNBC involve both genomic and epigenomic alterations. Therefore, to address the hypothesis that somatic mutations and epigenetic alterations regulate and impact gene regulatory networks and biological pathways driving TNBC and non-TNBC, we conducted pathway prediction and network analysis, focusing on genes harboring somatic mutations and epigenetic alterations unique to each type of breast cancer. Here we sought to: (i) unravel the genomic-epigenomic interaction landscape in TNBC and non-TNBC; (ii) gain insights about the broader biological context in which genomic and epigenomic factors operate; and (iii) to establish putative functional bridges between somatic mutations and epigenomic alterations and the pathways they affect. Such pathways could be potential therapeutic targets.

**Figure 5:**
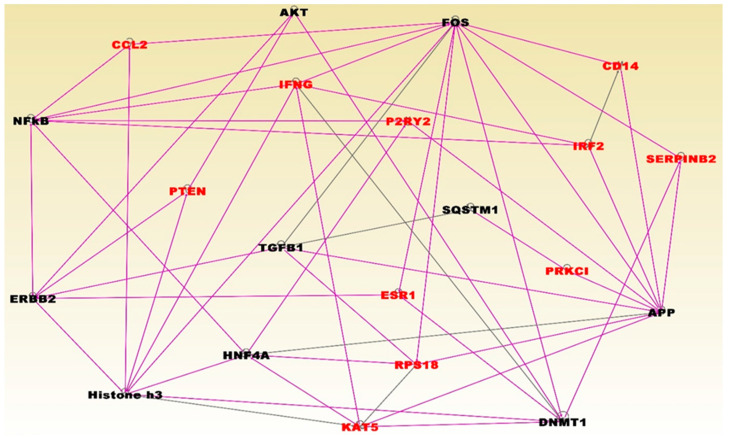
Molecular networks for genes enriched for somatic and epigenetic variation in TNBC. Genes are shown in nodes and vertices represent functional relationships. The gene symbols in red fonts represent genes containing somatic and epigenetic alterations in TNBC only. Genes in black font are other genes predicted to interact with genes containing somatic and epigenetic alterations. Note that, because the analysis involved merging the top networks containing genes with overlapping functions, the colors of the lines (vertices) are also overlapping to reflect this. Pink and back lines represent interactions among somatic and epigenetic altered genes (red fonts), and with predicted genes in black fonts.

The results of network and pathway analysis for the top most significant networks for TNBC are presented in Figure 5. The highest scoring networks included the genes predicted to be involved in: hereditary diseases, organismal injury and abnormalities (Z-score = 35); cellular development, cellular growth, proliferation, protein synthesis (Z-score = 30); cell-to-cell signaling and interaction, immune cell trafficking, cellular development, cellular growth and proliferation, embryonic development (Z-Score = 28); cellular assembly and organization, cellular compromise, DNA replication, recombination, and repair (Z-Score = 25); and cell cycle, cellular assembly and organization, molecular transport (Z-Score = 21). Network analysis revealed functional relationships and interactions among aberrantly methylated genes containing somatic mutations in gene regulatory networks (see symbols in red fonts) for TNBC (Figure 5). Network analysis also revealed functional relationships and interactions with other genes (see black fonts) (Figure 5).

**Figure 6:**
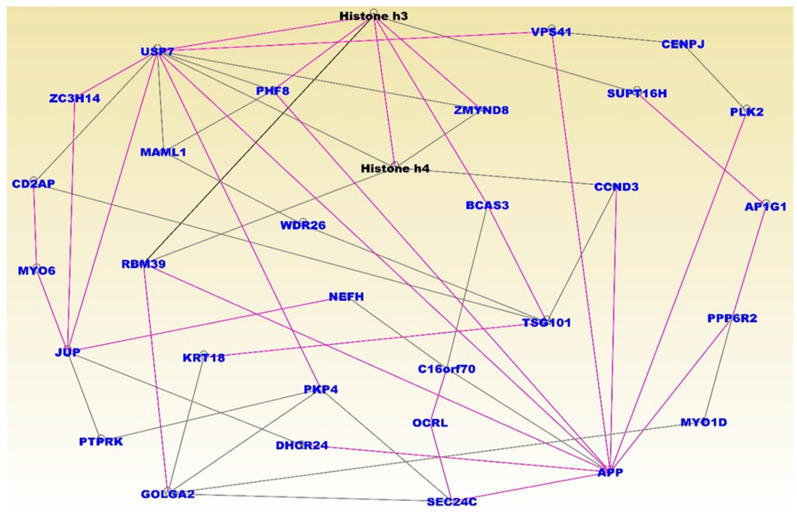
Molecular networks enriched for somatic and epigenetic variation in non-TNBC. Genes are shown in nodes, and vertices represent functional relationships. The gene symbols in blue fonts represent genes containing somatic and epigenetic alterations in non-TNBC only. Note that the use of blue fonts for somatic and epigenomic altered genes here was designed to provide a contrast between the two types of breast cancer. Genes in black fonts are other genes predicted to interact with genes containing somatic and epigenetic alterations. Note that, because the analysis involved merging the top networks containing genes with overlapping functions, the colors of the lines (vertices) are also overlapping to reflect this. Pink and back lines represent interactions among somatic and epigenetic altered genes (blue fonts) and with predicted genes in black fonts.

The somatic mutated genes that were also aberrantly methylated and interacting in gene regulatory networks included the genes CCL2, PTEN, ESR1, IFNG, P2RY2, INF2, CD44, SERPINB2, PRKC1, RPB18 and KAT5 (Figure 5). Pathway analysis revealed important pathways implicated in TNBC. The top five most significant pathways enriched for somatic and epigenetic variation included the retinal biosynthesis, BAG2, LXR/RXR, EIF2 and P2Y purigenic receptor signaling pathways. The results of network and pathway analysis for the topmost significant networks for non-TNBC are presented in Figure 6. The highest scoring networks included the genes predicted to be involved in: cellular assembly and organization and cellular compromise (Z-Score 59); cancer, organismal injury and abnormalities (Z-Score = 51); cell morphology and embryonic development, (Z-Score = 48); connective tissue disorders, organismal injury and abnormalities and organ development (Z-Score = 46); cellular compromise, cellular function and maintenance, cancer, (Z-Score 32); cancer and immunological disease (Z-Score = 28); cancer, cell death and survival, cellular development (Z-Score = 26). Network analysis revealed functional relationships and interactions among aberrantly methylated genes containing somatic mutations in gene regulatory networks (see symbols in red fonts) for non-TNBC (Figure 6). Network analysis also revealed functional relationships and interactions with other genes, including histone H3 and H4, which are epigenetically altered (see black fonts) (Figure 6).

The somatic mutated aberrantly methylated genes interacting in gene regulatory networks included the genes USP7, ZC3H14, CD2AP, MYO6, JUP, PTPRK, GOLGA2, PHF8, MAML1, RBM39, KRT18, PKP4, DHCR24, WDR26, NEFH, C16orf70, OCRL, SEC24C, AFP, TSG101, BCAS3, CCND3, ZMYND8, VPS41, CENPJ, SUPT16H, PLK2, AP1G1, PPP6R2 and MYO1D, which are represented in blue fonts (Figure 6). Pathway analysis revealed important signaling pathways implicated in non-TNBC. The most significant pathways included the UVB-Induced MAPK, PCP, Apelin endothelial, Endoplasmatic reticulum stress and mechanisms of viral exit from host signaling pathways. Overall, integrative analysis revealed gene regulatory networks and signaling pathways enriched for somatic mutations and epigenomic alterations, unique to each type of breast cancer. The analysis also demonstrated that a subset of genes altered in the tumor genome, transcriptome and the methylome distinguished TNBC from non-TNBC. Both within type of breast cancer and comparison analysis of gene expression and DNA methylation between the two types of breast cancer showed that DNA methylation affects gene expression in TNBC and non-TNBC, and that these effects differ between the two types of breast cancers, consistent with our previous report [22].

## 4. Discussion

Since the completion of TCGA [19], large omics data sets have been made available. With the use of these data resources, we are now well-positioned to understand the molecular mechanisms underlying the etiological and biological differences and drivers of the two types of cancers. Here, we performed integrative data analysis combining multi-omics data for the discovery of integrated genomic, epigenomic and gene expression profiling signatures, networks and signaling pathways unique to TNBC and non-TNBC, and to discover signatures distinguishing the two types of breast cancer. The discovery of somatic mutated genes, which are also aberrantly methylated, suggests that genes driving tumorigenesis in TNBC and non-TNBC are under both genomic and epigenomic control. Here, we discuss the potential translational impact and clinical significance of the results of this investigation below.

### 4.1. Novelty and Innovation of the Study

Our approach combined somatic, DNA methylation and gene expression data for the discovery of gene, somatic mutation and DNA methylation signatures unique to TNBC and non-TNBC and signatures, distinguishing the two types of breast cancer. Our approach represents a novel and unified approach to the discovery of potential clinically actionable biomarkers and targets for TNBC and non-TNBC, using multi-omics data. Discovery of integrated somatic mutation and DNA methylation signatures distinguishing the two types of breast cancer suggests that TNBC and non-TNBC may be amenable to mutation and DNA methylation-based classification. The discovery of signatures unique to TNBC and non-TNBC suggests that different biological mechanisms may be controlling the development and progression of each type of cancer. To our knowledge, this is the first study to combine three pieces of information (somatic, epigenetic and gene expression data) for the discovery of gene signatures unique to TNBC and non-TNBC, and signatures distinguishing the two types of breast cancer. Gene expression profiling to identify key differentially expressed genes and predict clinical outcomes and analysis of differences in serum protein mass spectrometry between TNBC and non-TNBC have been reported [35,36]. Breast cancer subtype identification using somatic mutations and DNA methylation have also been reported [37,38]. The novel and innovative aspect of this investigation is that it combined three types of omics data (somatic, epigenomic and gene expression variation) to identify signatures unique to TNBC and non-TNBC, and signatures distinguishing the two types of breast. This novel integrated genomics approach to discovery of potential clinically actionable molecular markers and potential targets for the development of novel therapeutics in TNBC and non-TNBC has not been previously reported.

### 4.2. Complementing Omics Data with Standard Clinical Protocols

Traditionally, there have been several widely accepted methods for clinical classification of types of breast cancer. These include classification based on: (i) histology using morphological characteristics; (ii) immunohistochemical (IHC) markers, also used here to guide data analysis; and (iii) more recently, using gene expression profiles as the standard [8,10,35]. The significance of our investigation is that these traditional protocols could be complemented by the results in this investigation. Integrating somatic, epigenomic and gene expression data with traditional protocols holds promise not only for defining the types of breast cancer, but also for causally associating such types with the molecular mechanisms driving and distinguishing them. Moreover, integrating omics with standard protocols may help to eliminate errors associated with traditional protocols, such as IHC [8,10] and improve patient classification.

### 4.3. Clinical Significance of Integrating Gene and Epigenomic Signatures in TNBC and Non-TNBC

Over the last decade, gene expression has gained prominence as the standard protocol in breast cancer [15,16,17,18]. Clinically validated prognostic gene signatures, such as PAM50 [15,16] and MammaPrint [17,18], are poised to improve clinical outcomes via precision medicine. One limitation and critical unmet need in the use of such assays is that they lack information on molecular drivers of the disease and biological factors regulating the tumor microenvironment. Given that somatic mutations drive tumorigenesis, enduring epigenetic landmarks define the tumor microenvironment [39], and DNA methylation affects gene expression [22], integrating information on gene expression, somatic mutation and DNA methylation, as demonstrated in this study, which provides an avenue for addressing this limitation. Although we did not explore the therapeutic potential of the discovered epigenomic markers in this study, several studies have reported the therapeutic potential and prognostic value of DNA methylation markers in breast cancer, specifically TNBC [40,41,42,43]. This is significant, because currently there are no effective targeted therapies for TNBC.

### 4.4. Somatic Mutation Signatures in TNBC and Non-TNBC

The discovery of somatic mutation signatures unique to TNBC and non-TNBC and the differences in somatic mutation burden between the two types of breast cancer is of particular interest, because somatic driver mutations confer selective clonal growth advantage to tumor cells. Importantly, the discovery of a signature of somatic mutated genes distinguishing TNBC from non-TNBC suggests that the two types of breast cancer may be amenable to mutation-based classification. This could enable the identification of patients at high risk of developing aggressive tumors, such as TNBC, who could be prioritized for treatment. In this study, we did not investigate the mutation-based classification of the two subtypes of breast cancer. However, cancer subtype identification using somatic mutation data has been reported [37], though our study focuses on TNBC and non-TNBC, a problem which has not been addressed.

### 4.5. Signaling Pathways as Potential Therapeutic Targets

Our investigation revealed biological pathways dysregulated by somatic mutations and aberrant DNA methylation, including retinal biosynthesis, BAG2, LXR/RXR signaling pathways in TNBC, which have the potential to serve as therapeutic targets if validated. The discovery of retinal biosynthesis signaling pathway is significant. This is because a major obstacle in the use of retinoid therapy in cancer treatment is tumor resistance to this agent [44]. One approach to overcoming the resistance of tumor cells to retinoic acid may be genomic and epigenomic reprogramming, targeting and suppressing this pathway using markers that have been shown to restore sensitivity to retinoic acid [44]. For example, curcumin has been shown to restore sensitivity to retinoic acid in TNBC cells [44], and to induce retinoic mediated apoptosis in retinoic acid resistant breast cancer cells [45]. Moreover, DNA methylation investigated here has been shown to predict the response of TNBC to all-trans retinoic acid [40]. This is particularly important, because retinoid signaling regulates breast cancer stem cell differentiation—a critical biological process in tumor development and progression [46].

Another pathway implicated in breast cancer discovered here was BAG2. The significance of this finding is that the Bcl-2-associated athanogene 2 (BAG2) has been shown to regulate the oncogenic functions of pro-cathepsin B in metastatic TNBC [47]. This makes the BAG2 discovered here a potential therapeutic target, which may reduce the burden of metastatic TNBC. We also discovered the LXL/RXR signaling pathway, which has been implicated in TNBC [48,49]. This is particularly interesting, because while our study was based on TCGA data, which is almost exclusively derived from women of European ancestry, the LXR/RXR signaling pathway has also been associated with TNBC in African American women using a different data set [49]. The significance of this finding is that African American women tend to have higher incidences and higher mortality rates from TNBC, and are affected at younger age—before the recommended age of 50 for screening [49].

We discovered signaling pathways associated with non-TNBC, notably the UVB-induced MAPK and apelin endothelial signaling pathways. The clinical significance of these findings is that the UVB-induced MAPK signaling pathway has been associated with estrogenic chemicals, most of which are considered carcinogenic [50]. Another pathway of clinical significance that we discovered in non-TNBC is the apelin signaling pathway [51,52,53]. This pathway was recently patented as a therapeutic target for breast cancer [51,52,53]. Taken together, these observations suggest that if confirmed, the discovered signaling pathways have therapeutic potential in TNBC and non-TNBC.

Apart from the discovered pathways, we also discovered a genomically and epigenomically altered ESR1 gene—a critical biomarker to both TNBC and non-TNBC. A negative ESR1, along with negative PR and lack amplification of HER-2, define TNBC. However, ESR1 also plays a major role in non-TNBC. Perhaps one of the most distinguishing features relevant to this investigation is that patients with estrogen receptor or hormone receptor-positive tumors (herein defined as non-TNBC) receive endocrine and targeted therapies [54], whereas TNBC is non-responsive, and there are currently no Food and Drug Administration [FDA] targeted therapies for TNBC. In a comprehensive review of the factors predictive of response to hormone therapy in breast cancer, Rastelli and Crispino confirmed the role of estrogen receptor content as a predictor of response to endocrine treatment [55]. They found that the benefits from endocrine treatment were directly proportional to estrogen receptor levels in non-TNBC [55]. Taken together, the discovery of a somatic mutated and epigenetic altered ESR1 in this study suggests that it could have potential therapeutic implications.

In sum, this investigation revealed that TNBC and non-TNBC are complex diseases, whose development and progression involve oncogenic interactions between somatic and epigenomic alterations, unique to each type of breast cancer. Our investigation further reveals that oncogenic interactions between these biological factors likely lead to the dysregulation of the gene regulatory networks and signaling pathways that drive TNBC and non-TNBC. We argue that integrating large-scale, high-dimensional somatic mutation, DNA methylation and gene expression data holds promise, not only for unraveling the genomic-epigenomic interaction landscape of TNBC and non-TNBC, but also provides the avenue for uncovering the molecular mechanisms characterizing the biological differences and the drivers of the two types of breast cancer.

Limitations: We have shown that integrative data analysis, combing information on somatic mutations, DNA methylation and gene expression, has the promise to unravel the genomic-epigenomic interaction landscape of TNBC and non-TNBC. However, the limitations of the study must be acknowledged. We used TCGA data, which is unique and optimal for integrative data analysis using disparate omics data from the same patient cohort. One key limitation worth mentioning here is that we did not use only driver somatic mutations in this investigation. Therefore, some of the somatic mutations used here may be passenger mutations, which do not contribute to TNBC or non-TNBC development. However, while passenger mutations may not contribute to disease development, they bear the imprints of the mutational mechanisms that have generated them by the process of natural selection. Therefore, they provide insights into the etiology and pathogenesis of TNBC and non-TNBC. Moreover, passenger mutations have been shown to accurately classify human tumors [56].

Another limitation worth noting here is that, in this study, we focused on TNBC and non-TNBC. Both types of breast cancer are inherently heterogeneous, each comprising of many subtypes not investigated here. Subtyping mapping was beyond the scope of this investigation, but is worth pursuing in future.

## 5. Conclusions

We discovered somatic, DNA methylation and gene expression signatures unique to TNBC and non-TNBC, and signatures distinguishing the two types of breast cancer. In addition, we discovered gene regulatory networks and signaling pathways enriched for somatic and epigenetic alterations in TNBC and non-TNBC. There were significant variations in patterns of mutation, DNA and gene expression profiles, reflecting the heterogeneity inherent in the two types of breast cancer. The study demonstrates that integrative analysis of multi-omics data is a powerful approach to unravelling the genomic-epigenomic interaction landscape, and for the discovery of potential clinically actionable biomarkers, and targets for the development of therapeutic targets for TNBC and non-TNBC. Further research is recommended to map the genomic and epigenomic interactions in the subtypes of the two types of breast cancers to facilitate precision medicine.

Data Sharing: Availability of data and materials used in this study. The data that support the findings of this study are provided in supplementary tables as documented below, and original data sets are also made available in the TCGA, and are downloadable via the Genomics Data Commons https://gdc.cancer.gov/.

## Figures and Tables

**Table 1:** Characteristics and distribution of samples, probes and gene symbols for the original data sets on gene expression, somatic mutation and DNA methylation for triple negative breast cancer (TNBC) and non-triple negative breast cancer (non-TNBC) used in this study.

Data Type	TNBC		Non-TNBC		Control
	Genes or Probes	Tumor Samples	Genes or Probes	Tumor Samples	Samples
Gene Expression	60,484 probes	110	60,484 probes	605	113
Methylation	485,578 probes	83	485,578 probes	597	83
Somatic Mutated	7659 genes	110	16,752 genes	605	-

**Table 2:** List of top 30 most highly significantly differentially expressed genes, which were also significantly differentially methylated distinguishing TNBC from non-TNBC, along with number of DNA methylation (DM) sites per gene and estimates of differential expression and differential methylation *p*-values. All *p*-values were adjusted for the false discovery rates.

Gene_Symbol	Chromosome Position	EXP-Value	DM_Sites	DM *p*-Value
*PTPRN2*	7q36.3	1.08 × 10^−15^	487	1.69 × 10^−19^
*PCDHGA1*	5q31	4.49 × 10^−2^	242	1.36 × 10^−22^
*MSLN*	16p13.3	2.86 × 10^−8^	165	1.50 × 10^−24^
*CBFA2T3*	16q24.3	8.05 × 10^−12^	146	4.07 × 10^−25^
*CUX1*	7q22.1	1.01 × 10^−3^	120	8.13 × 10^−28^
*ANKRD11*	16q24.3	2.92 × 10^−5^	114	3.40 × 10^−21^
*NCOR2*	12q24.31	1.97 × 10^−2^	112	1.07 × 10^−27^
*LMF1*	16p13.3	1.62 × 10^−14^	111	9.23 × 10^−27^
*ABR*	17p13.3	1.32 × 10^−3^	103	1.39 × 10^−24^
*SLC45A4*	8q24.3	1.12 × 10^−4^	103	3.29 × 10^−31^
*SYNGAP1*	6p21.32	5.78 × 10^−6^	99	2.34 × 10^−20^
*TP73*	1p36.32	2.26 × 10^−6^	96	2.27 × 10^−25^
*PRKAR1B*	7p22.3	9.45 × 10^−3^	95	3.32 × 10^−27^
*ADAMTS2*	5q35.3	1.47 × 10^−2^	86	1.07 × 10^−28^
*PRRT1*	6p21.32	3.99 × 10^−7^	86	1.63 × 10^−26^
*PCDHA2*	5q31.3	1.09 × 10^−3^	83	2.04 × 10^−10^
*ANK1*	8p11.21	5.84 × 10^−3^	82	1.75 × 10^−25^
*TBC1D16*	17q25.3	5.65 × 10^−3^	81	3.31 × 10^−31^
*HOOK2*	19p13.13	1.31 × 10^−4^	79	3.86 × 10^−23^
*COL23A1*	5q35.3	2.15 × 10^−5^	75	1.10 × 10^−34^
*NFATC1*	18q23	1.38 × 10^−2^	75	9.25 × 10^−33^
*FOXK1*	7p22.1	2.74 × 10^−5^	73	4.24 × 10^−33^
*BCOR*	Xp11.4	1.09 × 10^−7^	72	2.95 × 10^−21^
*COL4A2*	13q34	3.03 × 10^−2^	72	5.02 × 10^−25^
*PCDHA4*	5q31.3	2.16 × 10^−5^	72	2.04 × 10^−10^
*RGS12*	4p16.3	3.48 × 10^−8^	71	1.21 × 10^−31^
*RGL2*	6p21.32	1.31 × 10^−14^	69	4.34 × 10^−29^
*DNAH17*	17q25.3	1.22 × 10^−4^	68	1.74 × 10^−26^
*CTBP2*	10q26.13	1.51 × 10^−4^	67	6.16 × 10^−26^
*HOXC4*	12q13.13	1.60 × 10^−9^	67	3.63 × 10^−11^

**Table 3:** List of 30 somatic mutated genes significantly differentially expressed (DE), and also significantly differentially methylated (DM), between TNBC and non-TNBC.

Gene Symbol	Chromosome	DE *p*-Value	DM *p*-Value	DM Sites	TNBC Somatic Events	Non-TNBC Somatic Events
*RBM22*	5q33.1	2.21 × 10^−10^	5.04 × 10^−10^	7	3	
*CRIP1*	14q32.33	2.75 × 10^−22^	4.17 × 10^−27^	17	2	
*CYP51A1*	7q21.2	8.95 × 10^−5^	1.39 × 10^−11^	16	2	
*FAM227A*	22q13.1	3.39 × 10^−3^	5.00 × 10^−4^	5	2	
*FHL3*	1p34.3	3.32 × 10^−5^	1.11 × 10^−15^	9	2	
*GFI1*	1p22.1	7.77 × 10^−5^	4.41 × 10^−30^	63	2	
*HCP5*	6p21.33	2.04 × 10^−3^	6.76 × 10^−10^	39	2	
*HOXD13*	2q31.1	6.69 × 10^−7^	5.98 × 10^−21^	9	2	
*IGSF21*	1p36.13	2.82 × 10^−7^	6.04 × 10^−28^	50	2	
*P2RY2*	11q13.4	3.33 × 10^−12^	1.59 × 10^−19^	15	2	
*PCBP3*	21q22.3	1.64 × 10^−8^	9.13 × 10^−24^	42	2	
*RBM17*	10p15.1	3.11 × 10^−15^	3.98 × 10^−8^	6	2	
*RNF26*	11q23	1.99 × 10^−3^	1.28 × 10^−4^	4	2	
*SCAMP2*	15q24.1	8.94 × 10^−9^	1.15 × 10^−9^	4	2	
*SCNN1B*	16p12.2	3.39 × 10^−2^	4.08 × 10^−17^	9	2	
*MAP2K4*	17p12	1.66 × 10^−8^	2.73 × 10^−12^	7		33
*ZSWIM8*	10q22.2	1.19 × 10^−4^	3.34 × 10^−9^	4		18
*MALAT1*	11q13.1	2.28 × 10^−11^	3.79 × 10^−5^	4		16
*MED23*	6q23.2	1.13 × 10^−3^	8.65 × 10^−6^	9		16
*NRCAM*	7q31.1	3.22 × 10^−4^	1.08 × 10^−29^	17		16
*RAB3GAP2*	1q41	3.04 × 10^−3^	9.49 × 10^−15^	8		16
*KIAA0100*	17q11.2	1.13 × 10^−6^	3.46 × 10^−7^	5		15
*ABCC4*	13q32.1	8.68 × 10^−8^	1.04 × 10^−18^	17		13
*MED13L*	12q24.21	1.46 × 10^−13^	7.41 × 10^−23^	12		13
*RIPK1*	6p25.2	1.87 × 10^−6^	3.25 × 10^−14^	10		13
*TIAM2*	6q25.2-q25.3	1.83 × 10^−4^	1.67 × 10^−23^	36		13
*ADAMTS16*	5p15.32	2.51 × 10^−4^	6.92 × 10^−17^	32		12
*ADGB*	6q24.3	5.90 × 10^−12^	1.05 × 10^−3^	5		12
*PHF8*	Xp11.22	7.01 × 10^−7^	4.42 × 10^−8^	6		12
*RGS7*	1q43	1.30 × 10^−4^	2.95 × 10^−18^	26		12

## Supplementary Materials

The following are available online at https://www.mdpi.com/2072-6694/12/6/1559/s1, **Table S1:** A complete list of somatic mutated genes and somatic mutations in TNBC and non-TNBC, **Table SE:** A complete list of genes significantly differentially expressed between TNBC and controls and between non-TNBC and controls along with their estimates of *p*-values, **Table SM:** A complete list of genes significantly differentially methylated between TNBC and controls and between non-TNBC and controls along with their estimates of *p*-values, **Table S2A:** A complete list of all the 12,816 significantly differentially expressed genes which were also differentially methylated in TNBC, **Table S2B:** A complete list of all the 13,460 significantly differentially expressed genes which were also differentially methylated in non-TNBC, **Table S2C:** A complete list of all the 10,365 genes significantly differentially expressed also significantly differentially methylated in TNBC and non-TNBC, **Table S3A:** A complete list of all the 4400 significantly differentially expressed genes which were also significantly differentially methylated distinguishing TNBC from non-TNBC, **Table S3B:** A full list of all the 5663 genes with binary pattern of presence, **Table S4:** A complete list of somatic mutated genes significantly differentially expressed (DE) which were also significantly differentially methylated between TNBC and non-TNBC.
